# Managing patients with advisory defibrillator leads: what can we learn from published data?

**DOI:** 10.1007/s12471-015-0669-6

**Published:** 2015-03-12

**Authors:** F. A. Bracke, B. M. van Gelder

**Affiliations:** Department of Cardiology, Catharina Hospital, Michelangelolaan 2, 5623 EJ Eindhoven, The Netherlands

**Keywords:** Defibrillators, Implantable, Electrodes, Implanted, Complications, Advisory leads

## Abstract

Defibrillator lead advisories stir a lot of emotions, both with patients and physicians, and this may influence lead management. We reviewed the literature for a more evidence-based approach to this issue.

From the complications of two of the current advisory leads, the Medtronic Sprint Fidelis and St. Jude Riata leads, and the consequences of possible interventions, we can conclude that a restrained approach to premature replacement is appropriate. It may be opportune to replace the leads during a scheduled generator replacement in case of a higher electrical failure rate, in order to prevent future premature interventions.

We found no support to extract non-functional advisory leads. In contrast, extraction is often more demanding than anticipated, and the risk substantially exceeds that of simply abandoning the leads.

## Introduction

Lead advisories stir a lot of emotion and provoke impulsive responses. However, the Telectronics Accufix lead experience has shown that such a response did produce more fatal complications than the injuries related to the broken retention wire [1]. Moreover, lead-related complications occurred less frequently than originally anticipated.

The recent advisories of the Medtronic Fidelis (Medtronic, Minneapolis, Minnesota) and St. Jude Riata (St. Jude Medical, St. Paul, Minnesota) defibrillator leads again emphasise the need to guide management through objective information. Many patients with advisory leads are approaching generator replacement, and one has to decide whether to maintain the advisory lead operational, or to implant a new lead. In addition, a decision should be made between extracting or abandoning non-functional advisory leads. The ‘Recommendations from the Heart Rhythm Society Task Force on Lead Performance Policies and Guidelines’ lists factors that might influence these decisions [2]. Patient-related factors include pacemaker dependency, risk of life-threatening arrhythmias or of surgical revision, time to generator replacement, and psychological wellbeing. The considered lead characteristics are failure rate, predictability and consequences of failure, and means to prevent the latter by reprogramming the device. In this paper, we take a look at the literature to arrive at an informed approach to patients with advisory leads.

## Incidence of failure of contemporary advisory leads

To establish the electrical failure rate is not as straightforward as might be expected. The definition of electrical failure varies significantly, and consequently the reported incidence. This is amplified by disparities between cross-sectional and prospective studies, and between different durations of follow-up (it takes years before failure rates diverge from other leads). In case of Riata, the failure rate may also differ between subtypes: Abdelhadi et al. reported different electrical failure rates in 1081 patients between the 8F and ST 7F leads of 8 and 1.6 %, respectively, but with a different median follow-up of 4.2 and 3.3 years [3]. Lastly there is publication bias, as initially more extreme outcomes are published [4].

Publications from larger cohorts approximate more closely the Riata lead failure rate. Parkash et al. followed 4358 Riata leads with a median dwell time of 5.0 years, and found 5.2 and 3.3 % electrical failures in the 8F and ST 7F subtypes, respectively [5]. Hayes and colleagues prospectively followed 776 patients during 9.8 ± 2.0 months of follow-up, and found 1.3 % electrical lead failure with no significant differences between Riata 8F and ST 7F leads [4]. These failure rates are actually in line with historic defibrillator lead performance [6, 7].

The externalisation of the conductors in Riata leads should not be confused with electrical dysfunction, as most researchers found the latter to be independent of externalisation [4, 5, 8]. Only Theuns et al. registered more electrical failures with externalised conductors (10.9 vs. 3.5 %) [9]. However, in absolute numbers, the majority of failures occur in leads without externalised conductors.

The Medtronic Fidelis electrical failure rate is higher than the Riata failure rate: 9.1 % after 5 years in the 21,500 patients followed in the Medtronic Care Link PLUS data, and 16.8 % during the same follow-up in a Canadian cohort of 3126 patients [10, 11].

## Mortality and lead failure

It may be obvious that lead failure is not equivalent to mortality, but it is surprising that quantification of mortality seems to be elusive, even if more than 200,000 Fidelis and Riata leads each have been implanted. Hauser et al. screened the FDA Maude database (a databank of voluntary reports of device malfunctions), and found 22 patients in whom the demise could be linked with Riata lead failure [12]. However, the same report also mentioned five Medtronic Sprint Quattro leads related to mortality. The lack of a denominator in these case reports obscures the real incidence of mortality, as voluntary reporting may be biased towards more scrutiny for leads under advisory.

Only a single comparative study addressed the survival of patients with advisory leads vs. other leads, but found no difference in adjusted survival between the Medtronic Fidelis or the Sprint Quattro lead [13]. Moreover, in spite of the many publications on both Fidelis and Riata leads, mortality attributed to lead failure is seldom observed: only Parkash et al. mentioned one death out of 4358 Riata patients related to lead failure [5]. It should be noted that until now no mortality has been linked with conductor externalisation of Riata leads. In practice, the true mortality rate of advisory leads may remain elusive, as it would require systematic post-mortem interrogation of devices to separate it from the substantial natural attrition in a typical ICD population.

Only patients who would die in the absence of a working defibrillator are at risk from lead failure: in populations similar to the SCD-HeFT and MADIT II cohorts, this would account for 2–3 % every year (6 % mortality reduction in SCD-HeFT during a 3-year follow up, and 6.4 % in MADIT II after 2 years) [14–16]. In case of Fidelis leads and assuming a lead failure rate of 3 % per year, the natural attrition of around 5 % per year, and malfunctioning leads being replaced after routine controls or inappropriate shocks, some approximating calculus will show that the mortality risk is less than 0.1 % per year [15–17].

## Morbidity

The most debilitating complication of advisory lead failure is inappropriate shocks from lead noise that is considered to be an arrhythmia by the ICD. But this is not exclusive for recalled leads, as these shocks occurred even with conservative programming in the first year after implant in 3.6 and 3.0 %, respectively, of patients in the PREPARE and MADIT-RIT study [18, 19]. A single-centre Dutch study following 1075 implants reported a 3 % incidence after 31 ± 17 months of follow-up [20]. In comparison, even though the mode of presentation in about half of the defective Fidelis or Riata leads is inappropriate shocks, the absolute incidence ranges from only 0.7 to 3.6 % of all implanted leads [5, 11, 21, 22].

In the PREPARE and MADIT-RIT studies, the cause of inappropriate shocks is mostly supraventricular tachycardia and not lead noise. It is therefore uncertain whether lead-related inappropriate shocks have the same impact on survival as in these studies [19, 23].

Inappropriate shocks can have a profound influence on the psychological wellbeing of the patient. There is a significant relation in the general ICD population between procedure-related complications and anxiety, but not with depression [24]. Compared with patients with non-advisory leads, patients with uncomplicated advisory leads show no difference in psychological functioning [25]. Notwithstanding the often dramatic presentation of inappropriate shocks in clusters and during full consciousness, literature is not unequivocal about the long-term influence on psychological wellbeing. Actually, only a few small studies have addressed the impact on anxiety and depression. Only one out of five showed a significant difference in depression scores, and a minority of seven studies a small to moderate difference in anxiety scores between shocked and non-shocked patients [26].

The activation of lead integrity alerts and programming longer detection intervals have significantly reduced the incidence of inappropriate shocks. Kallinen et al. reported a reduction of shocks from 69 to 17 %, and of the average number of shocks in a cluster from 13 to 3, and Swerdlow et al. a 46 % shock reduction and a more than 50 % reduction in clusters of more than 5 shocks [27, 28]. The PREPARE and MADIT-RIT trials have demonstrated that allowing more time for spontaneous termination of arrhythmia by programming longer detection parameters resulted in a substantial reduction of inappropriate therapy [18, 19]. However, implementing these interventions should be standard practice in every ICD patient.

Pacing dependent patients may be more directly affected by lead failure through inhibition of pacing from noise detection, or non-capture from lead fracture. Symptomatic inhibition of pacing or failure to capture is nonetheless only sporadically observed. Hauser et al. reported failure to pace in 13 out of 848 patients with a Fidelis lead. Only one patient experienced syncope but there was no mortality [29]. In a Canadian study, 11 out of 3169 patients with Fidelis leads had symptomatic inhibition of pacing, also without mortality [11]. This may seem reassuring at first sight, but patients without any intrinsic rhythm are probably more at risk of asystole in case of lead malfunction than of ventricular fibrillation at any time.

## Proactively replacing advisory leads

Dysfunctional leads are to be replaced when continuation of (reliable) defibrillator therapy is required. However, there are no studies that compare the preventive replacement of a still normally functioning advisory lead with a wait-and-see approach.

Adding any new lead, or even replacing the generator, is not without complications. The Danish pacemaker registry reported a 2.9 % infection rate after elective pacemaker generator exchange in 8380 patients [30]. Replacing advisory ICD generators in 533 patients in a Canadian study resulted in more complications than from device malfunction: 5.8 % major complications, including two deaths from lead extraction because of infection [31]. Also Costea et al. reported 4.1 % major complications after replacing advisory generators [32].

The REPLACE study demonstrated that inserting an additional lead (excluding upgrades to cardiac resynchronisation therapy) had a major complication rate of 12.7 % in contrast to 4.9 % if only the generator was exchanged [33]. Eckstein et al. reported an eight-fold increase of lead complications after implanting new shock or pace-sense leads after lead failure when compared with only exchanging the generator [34]. Many of the premature interventions will also foreshorten the lifecycle of the generator as it is often concurrently replaced, and this adds to the cost of the therapy.

It may be more favourable to replace advisory leads during elective generator replacement, as only the additional risk of inserting a new lead has to be accounted for. Bashir et al. calculated the cost and effectiveness of a proactive lead replacement strategy at the time of an elective generator replacement for patients under 60 years, with normally functioning Sprint Fidelis leads, and an expected failure rate of 5.2 % per year as observed in their population [17]. Even when the cost of elective lead extraction in one-third of the procedures was included, they still prove that the proactive strategy was more cost-effective than waiting for lead failure to occur. Also 21 impromptu lead failures per 100 patients were avoided, and this limited morbidity and unscheduled interventions. This benefit is influenced by the incidence of failure, the ratio of leads extracted during replacements, and individual patient profiles.

Implanting a totally subcutaneous defibrillator or S-ICD in eligible patients could be an alternative as transvenous lead introduction is avoided. This approach introduces additional surgery, as the depleted generator still has to be removed. Moreover, on-going studies such as the PRAETORIAN trial have to prove that the complication rate including the incidence of inappropriate shocks, shock efficiency and mortality is on par with current transvenous ICD systems [35].

## Extracting or abandoning non-functional leads

The expert consensus of the Heart Rhythm Society on lead extraction states that non-functional leads that interfere with the operation of implanted devices have a class I indication for extraction, and non-functional leads that pose no immediate threat to the patient a class IIb indication [36]. Both have a level of evidence C, and this is also reflected by Maytin et al. who mentioned that the risk of abandoning non-functional leads is considered ‘to be real’ by most lead extraction experts, although the latter have to admit to the lack of evidence [37]. In the consensus paper it is noted that many important clinical questions have not yet been addressed by high-quality investigations, or do not lend themselves to experimentation. The expert opinion is based on the assumptions that removing leads is necessary to avoid electrical interference, to prevent accumulation of abandoned leads that predisposes to venous occlusion, and to avert more difficult future extractions from increasing dwell times.

In contrast to this, all published reports indicate the safety of abandoning non-functional leads. Interference with abandoned leads is in most cases easily avoided if integrated bipolar leads are not used to avoid contact between coils that may result in oversensing. Sung et al. observed no adverse events attributable to interaction between newly implanted and abandoned Riata leads in 29 patients [22]. Bohm et al. remarked that no complications occurred after they started securing the (pacemaker) leads to prevent migration, and capping them to avoid electrical interference [38]. Suga et al. followed 531 patients with abandoned pacing leads, of whom only 18 needed extraction: in 7 because of venous obstruction to gain vascular access and in 8 owing to infection [39]. Likewise, Glikson et al., Bode et al. and Amelot et al. did not find any clinically significant risk from abandoning defibrillator leads in 78, 60 and 37 patients, respectively [40–42].

There has never been proof of a link between venous obstruction and the number of indwelling leads, and lead extraction itself has not been cleared from predisposing to this complication [43, 44]. In fact, when advisory leads are replaced with leads with established low failure rates, only a minority of patients will need future additional leads. Thrombus formation has been reported with externalised conductors of a Riata lead, but this is not exclusive to this type of lead [45–47]. Although extreme externalisation of Riata leads has been suggested to be potentially dangerous, there are no reports of clinical complications from this phenomenon.

Venkataraman et al. calculated that extraction of all non-functional sterile leads in order to prevent venous occlusion compared with extraction reserved for patients who present with occlusion (with an estimated incidence of 5 %) would result in a ten-fold higher mortality, even considering a doubled mortality rate because of longer dwell times [48]. If a similar calculation is made for infection, with an estimated incidence between 2–3 % after any surgical intervention, preventive extraction will likewise have a higher mortality than when extraction is limited to infected leads [30, 36]. A conservative approach also avoids unnecessary extraction procedures in more than 95 % of patients, and hereby considerably reduces the cost of managing advisory leads: Mehrotra et al. calculated an additional cost of $ 12,000 to $ 13,000 per extraction procedure compared with abandoning the leads [49]. Bashir et al. calculated that extraction of two-thirds of the leads in the setting of proactive replacing advisory leads at the time of generator exchange would result in an incremental cost of $ 12,779 per ICD lead failure avoided [17].

Extraction of advisory leads is not as easy as often suggested. Specialised tools, such as laser sheaths, are frequently necessary to extract Fidelis leads notwithstanding relatively short dwell times: in 51 % of the extractions by Maytin et al. and in 33 % by Parkash et al. with implant times of a mean 28 months or less than 4 years, respectively [50, 51]. Extraction of Riata leads may be even more demanding, as breach of the insulation material by externalisation of the conductors may result in ‘snowploughing’ of the insulation material in front of the extraction sheaths. The 8F Riata shock coils also lack backfilling, which may result in more ingrowth of scar tissue. This correlates well with the need of powered extraction sheaths in 18 out of 20 Riata patients by Patel et al., and in 60 % of procedures by Maytin et al. (the latter with a mean dwell time of only 42 months) [37, 52]. In a mixed population of Fidelis and Riata, Brunner et al. needed powered sheaths in 83.7 % of 430 cases with a median implant time of less than 5 years [53].

Some authors suggest that the risk of extracting advisory leads is low provided it is executed in experienced centres and in patients without comorbidities, but this is not confirmed by the data. Parkash et al. experienced major complications in 8.1 % of patients including one death during extraction of a Fidelis lead [51]. Brunner et al. reported that safety and efficacy was comparable with that of a non-recalled ICD lead (0.4 % mortality in their centre), but they still experienced two fatal complications: one out of 121 Riata leads, and one out of 308 Fidelis leads [53, 54]. There is often no motivation provided why some advisory leads are extracted and others abandoned. Parkash et al. performed formal extraction of 248 Fidelis leads, but abandoned 51 leads after simple traction failed, and did not attempt extraction in 169 leads, but the authors did not discuss the indication [51]. In order to estimate the risk of lead extraction, the reader should not be deceived by the published overall results of lead extraction, as these are often dominated by much shorter implant times than current advisory leads.

## Conclusion

Although there is a potential risk of lethal complications from current advisory leads, there is no indication that it surpasses that of prematurely replacing these leads. However, a scheduled generator exchange may be an opportune moment to consider adding a new defibrillator lead with established reliability with a limited additional risk. Still, there are no data or guidelines on what exact lead failure rate justifies this approach.

The accumulative risk of lead failure during the service time of an ICD increases with the expected longer battery life of current devices. Together with the failure rate of the Fidelis leads, it favours lead replacement during a generator exchange in our opinion. In contrast, it may be less beneficial with the currently observed electrical defects of the Riata leads. Independent and prospective collection of data on lead performance is needed for continuing evaluation of changing risk profiles.

Individual patient profiles influence the approach: in elderly patients the risk of failure may be lower, time at risk shorter, and procedural risks higher. In contrast, frequent need for defibrillator therapy, true pacing dependency, or the psychological burden of living with an advisory lead, may incline us to replace the lead.

Finally, there is no support in the literature for preventive extraction of sterile, non-functional (advisory) leads. In contrast, lead extraction has a definite risk of mortality and morbidity that is absent when leads are properly abandoned.

### Funding

None.
